# Genetic Predictors of Comorbid Course of COVID-19 and MAFLD: A Comprehensive Analysis

**DOI:** 10.3390/v15081724

**Published:** 2023-08-12

**Authors:** Mykhailo Buchynskyi, Valentyn Oksenych, Iryna Kamyshna, Sandor G. Vari, Aleksandr Kamyshnyi

**Affiliations:** 1 Department of Microbiology, Virology, and Immunology, I. Horbachevsky Ternopil National Medical University, 46001 Ternopil, Ukraine; 2 Broegelmann Research Laboratory, Department of Clinical Science, University of Bergen, 5020 Bergen, Norway; 3 Department of Medical Rehabilitation, I. Horbachevsky Ternopil National Medical University, 46001 Ternopil, Ukraine; 4 International Research and Innovation in Medicine Program, Cedars–Sinai Medical Center, Los Angeles, CA 90048, USA

**Keywords:** MAFLD, COVID-19, NAFLD, SARS-CoV-2, PNPLA, rs738409, GCKR, rs780094, TM6SF2, rs58542926, LYPLAL1, rs12137855

## Abstract

Metabolic-associated fatty liver disease (MAFLD) and its potential impact on the severity of COVID-19 have gained significant attention during the pandemic. This review aimed to explore the genetic determinants associated with MAFLD, previously recognized as non-alcoholic fatty liver disease (NAFLD), and their potential influence on COVID-19 outcomes. Various genetic polymorphisms, including PNPLA3 (rs738409), GCKR (rs780094), TM6SF2 (rs58542926), and LYPLAL1 (rs12137855), have been investigated in relation to MAFLD susceptibility and progression. Genome-wide association studies and meta-analyses have revealed associations between these genetic variants and MAFLD risk, as well as their effects on lipid metabolism, glucose regulation, and liver function. Furthermore, emerging evidence suggests a possible connection between these MAFLD-associated polymorphisms and the severity of COVID-19. Studies exploring the association between indicated genetic variants and COVID-19 outcomes have shown conflicting results. Some studies observed a potential protective effect of certain variants against severe COVID-19, while others reported no significant associations. This review highlights the importance of understanding the genetic determinants of MAFLD and its potential implications for COVID-19 outcomes. Further research is needed to elucidate the precise mechanisms linking these genetic variants to disease severity and to develop gene profiling tools for the early prediction of COVID-19 outcomes. If confirmed as determinants of disease severity, these genetic polymorphisms could aid in the identification of high-risk individuals and in improving the management of COVID-19.

## 1. Introduction

As of 18 June 2023, the number of global reported cases of severe acute respiratory syndrome coronavirus 2 (SARS-CoV-2) infection has exceeded 767 million, resulting in over 6.9 million fatalities [1]. The clinical manifestations of this infection range widely, encompassing mild or asymptomatic cases to severe acute respiratory syndrome.

However, it has become evident that the outcome of infection is heavily influenced by host-related factors, including advanced age [2,3,4,5], male gender [2,4,5], and the presence of various comorbidities such as hypertension [6,7], cardiovascular disease [5], obesity [2,3,8,9], and type 2 diabetes [5,10,11,12,13].

While the role of virally driven hyperinflammation, which leads to an excessive release of cytokines and triggers a phenomenon known as a “Cytokine storm,” remains a topic of controversy [14], the involvement of inflammatory processes in the severity of COVID-19, particularly among patients with comorbidities, is widely acknowledged [15].

Since the initial phase of the pandemic, the observation of familial clustering of severe COVID-19 cases has suggested the potential contribution of a genetic predisposition [16]. Therefore, it is plausible to consider that the intricate nature of the host’s genetic background, characterized by various polymorphisms, could significantly impact the pathogenesis and outcome of COVID-19. While advanced age, male sex, obesity, diabetes mellitus (DM), and other comorbidities have been established as risk factors for severe forms of the disease, these factors alone do not adequately explain the wide-ranging inter-individual variations observed in the severity of COVID-19 [17,18].

Therefore, the influence of genetic variations on clinical outcomes must be considered [18,19]. In this regard, multiple studies have elucidated the involvement of genetic polymorphisms in the susceptibility to and severity of COVID-19, with these polymorphisms being implicated in various biological pathways associated with the disease [18,19,20].

Interferons (IFN) serve as key mediators of antiviral signaling, stimulating the release of numerous vital components involved in the early host response to viral infection. Consequently, polymorphisms occurring in IFN genes or their receptors have been linked to an increased susceptibility to COVID-19 or more severe clinical outcomes [19,21,22].

Another crucial genetic mechanism involved in combating viral infections involves a genomic locus that harbors three genes responsible for encoding antiviral 2′,5′-oligoadenylate synthetase (OAS) enzymes (OAS1, OAS2, and OAS3). These enzymes are interferon-inducible antiviral proteins that activate the latent form of ribonuclease L (RNase L) [23,24]. Particularly, the RNase L pathway assumes specific significance in the immune response mounted against SARS-CoV-2, an RNA virus [19].

Regarding susceptibility to COVID-19, considerable attention has been directed toward investigating polymorphisms located in the angiotensin-converting enzyme 2 (ACE2) and transmembrane serine protease 2 (TMPRSS2) genes, which are directly involved in viral binding and the subsequent entry of the virus into host cells [25,26,27]

Non-alcoholic fatty liver disease (NAFLD), currently recognized as metabolic-associated fatty liver disease (MAFLD), encompasses a spectrum of conditions that range from simple steatosis with or without mild inflammation to a necroinflammatory subtype characterized by hepatocellular injury, known as non-alcoholic steatohepatitis (NASH), and eventual progression to cirrhosis [28,29]. MAFLD represents the most prevalent cause of chronic liver disease globally, with an estimated impact on approximately one-fourth of the global population [30,31]. The adoption of a novel definition for MAFLD has been proposed as a more suitable characterization of the hepatic manifestation of metabolic syndrome compared to the conventional definition of NAFLD [32,33,34].

While the relationship remains contentious, initial reports during the COVID-19 pandemic suggested that patients with MAFLD may face an elevated risk of experiencing a more severe disease course [35,36,37,38,39,40]. However, it remains unclear whether MAFLD merely associates with adverse outcomes or whether it plays a causal role [41]. Furthermore, it is crucial not only to acknowledge the potential impact of MAFLD on the course of COVID-19 but also to recognize the effects of the COVID-19 pandemic itself on patients with MAFLD and the overall epidemiology of the disease [41,42].

The pathogenesis of fatty liver is influenced by genetic factors as well. Notably, a large-scale genome-wide association study (GWAS) identified specific DNA sequence variants, including Patatin-like phospholipase domain-containing 3 (PNPLA3, rs738409-G), Glucokinase regulator (GCKR, rs780094-T), and Lysophospholipase-like 1 (LYPLAL1, rs12137855-C) that were associated with computed tomography-defined steatosis and biopsy-proven NAFLD characterized by lobular inflammation and fibrosis [43]. Moreover, other investigations have elucidated the functional significance of Transmembrane 6 superfamily member 2 (TM6SF2, rs58542926-T), a variant at the NCAN loci [44,45]. Among these genetic variants, PNPLA3 rs738409-G emerges as the most robust risk factor for NAFLD, exhibiting an odds ratio of 3.24 for histologic NAFLD [43]. Additionally, GCKR rs780094-T and TM6SF2 rs58542926-T have been recognized as significant determinants contributing to inter-individual variation in liver fat content [46,47,48,49]. Nevertheless, the functional implications of LYPLAL1 rs12137855-C remain relatively less explored.

**Figure 1:**
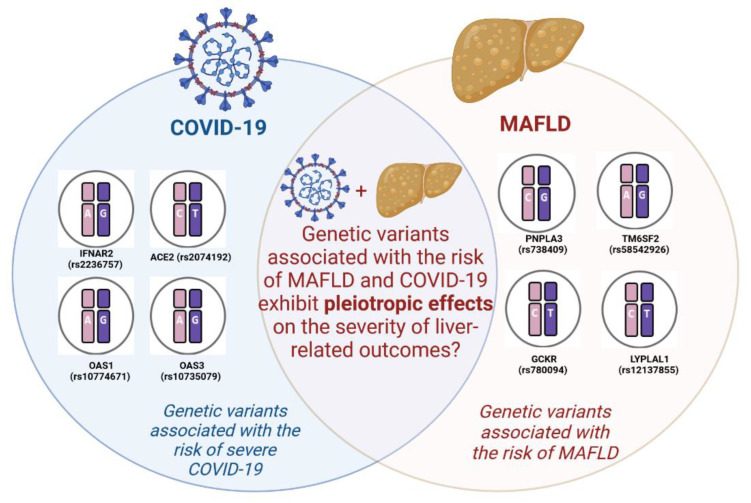
Genetic polymorphisms contributing to the risk of NAFLD and COVID-19.

Regarding the impact of these NAFLD-associated genetic polymorphisms on the course of COVID-19, the precise mechanisms remain incompletely understood (Figure 1). Nonetheless, several studies have already emerged, revealing unexpected associations between these genetic factors and COVID-19 outcomes [50,51,52,53].

## 2. Genetic Polymorphisms Associated with Susceptibility to COVID-19

### 2.1. ACE2

ACE2 serves as a transmembrane protein and functions as the principal entry receptor for certain coronaviruses, including SARS-CoV, MERS-CoV, and SARS-CoV-2, facilitating their entry into host cells [54]. The expression of ACE2 has been associated with an increased number of viral binding sites on cell membranes, rendering carriers susceptible to infection. In particular, the ACE2 single nucleotide polymorphism (SNP) rs2074192 has been identified as a risk factor for hypertension in adult males with obesity [55]. Moreover, rs2074192 has been implicated in the development of type 2 diabetes mellitus and cardiovascular disease [56].

The presence of the intronic variant rs2074192 has been associated with modifications in RNA secondary structure, which may disrupt the delicate equilibrium between ACE2 transcription and translation. This dysregulation has implications for the binding affinity of SARS-CoV-2 to angiotensin receptors [57]. Additionally, earlier studies have demonstrated that COVID-19 patients with coexisting hypertension experienced reduced mortality rates when treated with ACE inhibitors or angiotensin II receptor blockers (ARBs) compared to individuals who did not receive these medications [58].

ACE2 polymorphism (rs2074192) in obese, smoking males has been associated with greater variability in outcomes for COVID-19 disease, leading to more divergent outcomes [59].

Several investigations have highlighted a potential link between ACE2 gene polymorphisms and disease severity in individuals infected with SARS-CoV-2 (Table 1). Notably, a study conducted by Sienko et al. demonstrated a significant correlation between the ACE2 receptor gene rs2074192 polymorphism and the severity of COVID-19 in adult patients [60]. The authors observed a strong association between the ACE2 rs2074192 TT-genotype and adverse outcomes in patients with severe forms of COVID-19 (*p* = 0.0016). These findings align with a separate study conducted by Cafiero et al., which reported a higher prevalence of the T-allele of ACE2 rs2074192 in symptomatic individuals compared to asymptomatic Italian patients [61].

Ma et al. investigated the association between rs2074192 and COVID-19 in the Chinese population, revealing a significant relationship (*p* < 0.05) [62]. Additionally, Molina et al. found that the heterozygosity of rs2074192 SNPs in ACE2 was associated with disease severity caused by SARS-CoV-2, acting as a protective factor specifically in women.

Several studies have also reported conflicting results regarding the association between this ACE2 variant and disease outcomes [63,64,65]. These discrepancies in findings may be attributed to variations in sample sizes and the genetic backgrounds of the populations under investigation.

### 2.2. IFNAR2

Interferons (IFNs) encompass a diverse group of cytokines that elicit various biological activities through the induction of thousands of interferon-stimulated genes (ISGs). These ISGs exhibit antiviral, antiproliferative, antiangiogenic, and immunomodulatory functions [66]. The antiviral response is amplified and disseminated by innate IFN types I and III (IFN-α/β and IFN-γ, respectively). While IFN-λ mainly acts on mucosal epithelium due to receptor expression constraints, IFN-α/β exerts its effects on all nucleated cells, making it indispensable in the antiviral defense mechanism [67,68,69].

The genetic association studies conducted during the coronavirus disease 2019 (COVID-19) outbreak have highlighted the notable involvement of IFNAR2 (Table 1). Pairo-Castineira et al., in collaboration with a group of researchers, carried out an extensive genome-wide association study known as GenOMICC (genetics of mortality in critical care). This study encompassed a cohort of 2244 critically ill COVID-19 patients admitted to 208 intensive care units across the United Kingdom. The findings of this study demonstrated a significant association between the IFNAR2 rs2236757 gene variant and an increased severity of the disease [19].

Likewise, various studies employing diverse methodologies have corroborated the significance of IFNAR2 as a crucial gene implicated in the severity of COVID-19 [70,71,72,73,74].

In their research, Fricke-Galindo et al. (2022) discovered a notable association between the genetic polymorphisms of IFNAR2 (rs2236757, rs1051393, rs3153, rs2834158, and rs2229207) and an increased mortality risk in individuals afflicted with COVID-19 [21]. Intriguingly, the non-surviving group exhibited significantly lower levels of soluble receptors in comparison to the surviving group. These findings are consistent with previous studies that have elucidated the suppression of the IFN-I activation pathway by SARS-CoV-2, resulting in reduced levels of IFN-α and -β among COVID-19 patients [75,76,77]. Furthermore, the group of survivors displayed higher levels of sIFNAR2 in comparison to the non-survivors, indicating an augmented antiviral activity of IFN facilitated by the stability conferred by sIFNAR2 [21].

The findings from the investigation conducted by Dieter K. et al. (2022) [22] further support the existing body of research [19,21] by confirming the association between the rs2236757 genotype of IFNAR2 and an elevated risk of hospitalization in intensive care units and mortality among patients with COVID-19.

These results suggest that the rs2236757 polymorphism may contribute to reduced expression of IFNAR2, consequently predisposing individuals to more severe manifestations of COVID-19.

### 2.3. OAS

Following viral infection, the immune system initiates the production of antiviral cytokines, with interferons (IFNs) being particularly prominent. Among the IFN-stimulated genes, the 2′,5′-oligoadenylate synthetases (OAS) family plays a crucial role in the innate immune response. OAS proteins exhibit antiviral functions by serving as nucleotidyltransferases, facilitating the oligomerization of ATP into 2′,5-linked oligoadenylates (2-5A). This process leads to the activation of latent RNase L, which provides antiviral protection through the degradation of viral RNA [78,79,80,81]. The human OAS gene family comprises four genes, namely OAS1, OAS2, OAS3, and OAS-like (OASL), which are located on chromosome 12. Alternative splicing of these genes gives rise to 10 isoforms [82,83,84].

Several genome-wide association studies (GWASs) have identified various genetic variants at specific loci that are associated with susceptibility to COVID-19 (Table 1), either in general or in severe cases, when compared to controls from the general population [19,85].

One of the prominent variants identified within these loci is rs10774671 located at 12q24.13, which encompasses three genes responsible for encoding OAS enzymes, namely OAS1, OAS2, and OAS3 [19,85].

The investigation carried out by Banday et al. (2022) provided compelling evidence of the substantial influence of rs10774671 on the expression of OAS1, a crucial antiviral protein involved in the eradication of SARS-CoV-2, and its overall impact on the hospitalization outcomes of individuals with COVID-19 [86]. They propose that the functional impact of rs10774671 contributes to the association with COVID-19 severity by modulating the abundance of the OAS1 protein [86].

In a separate study, Pairo-Castineira et al. identified an association between the polymorphism OAS3 rs10735079 and the development of critical illness in individuals with COVID-19 [19].

**Table 1:** Summary of commonly reported single nucleotide polymorphisms associated with susceptibility to COVID-19.

Gene	SNP	Patient # **	SNP Effects	Significance	Features	Population	References
IFNAR2	rs2236757 G/A	694	Associated with severe forms of COVID-19 and increased mortality.	*p* = 0.031	In patients of non-white ethnicity, the presence of the A allele was linked to an increased risk of both intensive care unit (ICU *) admission and mortality.	Brazil population	[22]
		2244	Associated with severe forms of COVID-19.	*p* < 0.005	The A allele demonstrated an association with an elevated risk of developing severe COVID-19. Additionally, lower expression of IFNAR2 was observed in individuals with life-threatening cases of COVID-19.	UK population	[19]
		1202	Associated with mortality risk among patients with severe COVID-19.	*p* = 0.023	N/A ***	Mexico population	[21]
ACE2	rs2074192 G/A	318	Associated with the disease severity caused by SARS-CoV-2.	*p* = 0.016	Heterozygosity of rs2074192 was identified as a protective factor against COVID-19 infection in women.	Spain population	[87]
	rs2074192 C/T	104	Correlated with more severe outcomes of SARS-CoV-2 infection.	*p* = 0.0088 (for female) *p* < 0.0001 (for male)	The T-allele exhibited a higher prevalence in symptomatic patients compared to asymptomatic individuals.	Italian population	[61]
		293	Was not associated with COVID-19.	*p* > 0.005	The ACE2 rs2074192 variant does not confer a predisposition to the development of long COVID symptoms in individuals who were previously hospitalized due to COVID-19.	Spain population	[65]
		191	Was not associated with COVID-19	*p* > 0.005	N/A	China population	[64]
		481	Was not associated with COVID-19.	Severe: *p* = 0.49 Critical: *p* = 0.6	N/A	Mexico population	[63]
		456	Associated with COVID-19.	*p* < 0.001	Rs2074192 may potentially correlate with susceptibility to COVID-19-related cardiovascular complications and acute inflammatory infections.	China population	[62]
		188	Associated with an increased risk of a more severe disease course of SARS-CoV-2 infection.	*p* = 0.002	A strong correlation was observed between the TT-genotype of ACE2 rs2074192 and unfavorable outcomes in individuals with severe forms of COVID-19.		[60]
OAS1	rs10774671 A allele	3084	Associated with susceptibility to COVID-19.	Europeans: *p* < 0.005; Africans: *p* = 0.079	The presence of the A allele in rs10774671 may lead to a decreased expression of OAS1, thereby increasing the specific human risk of developing severe COVID-19.	European, Asian, African, African American and Hispanic populations	[86]
OAS3	rs10735079 A/G	2244	Associated with severe forms of COVID-19.	*p* < 0.001	N/A	UK population	[19]

* ICU—intensive care unit; ** #—number of patients; *** N/A—data not available.

## 3. Genetic Polymorphisms Associated with Susceptibility to MAFLD

### 3.1. PNPLA3

An integral aspect of MAFLD pathogenesis involves the perturbation of the lipid metabolism, resulting in the aberrant accumulation of lipids within the liver, specifically steatosis. The principal source of hepatic triglycerides (TG) arises from adipocyte-released free fatty acids (FFAs), which are facilitated by lipase enzymes [88,89]. Adipose triglyceride lipase (ATGL), encoded by the Patatin-like phospholipase domain-containing 2 (PNPLA2) gene, represents a significant enzyme involved in this cascade. In 2008, a pioneering genome-wide association study (GWAS) investigating NAFLD patients, encompassing diverse cohorts of Hispanic, African American, and European American people, revealed noteworthy findings [90]. Specifically, the presence of a genetic variant in Patatin-like phospholipase domain-containing 3 (PNPLA3), known as rs738409 or I148M, was linked to heightened lipid accumulation, even after adjusting for crucial factors such as ethnicity, body mass index (BMI), diabetes status, and alcohol consumption [90]. Furthermore, a range of studies conducted across distinct populations has further elucidated the impact of the I148M variant and other variants associated with NAFLD (Table 2).

Within the PNPLA3 gene, the production of adiponutrin, a triacylglycerol lipase, takes place, enabling the hydrolysis of triacylglycerols. However, the presence of the I148M variant has been shown to impede the enzymatic activity of this lipase, thereby fostering the onset of hepatic steatosis [91]. A recent meta-analysis investigating the impact of the I148M variant on NAFLD risk revealed that individuals carrying the minor G-allele had a 19% increased risk of developing NAFLD. Notably, the risk escalated to 105% among individuals harboring both GG alleles [92].

The I148M variant of PNPLA3 exhibits a comparable effect to more advanced stages of NAFLD. A meta-analysis encompassing 16 studies demonstrated that individuals carrying homozygous GG alleles have a 3.5-fold increased risk of developing NASH and a 3.2-fold elevated risk of experiencing fibrosis [93]. Furthermore, another meta-analysis revealed a significant association between the I148M variant and a 2.54-fold increased risk of NASH development [94]. Notably, a significant dose-dependent relationship of the G-allele was observed concerning this risk [92,94]. Similarly, an association between the I148M variant and cirrhosis progression was also noted. Specifically, the presence of a single G-allele conferred a 2-fold increased risk of developing cirrhosis, while individuals with homozygous GG alleles had a 3-fold higher risk compared to those with CC genotypes [95].

### 3.2. TM6SF2

The rs58542926 (E167K) variant, derived from the Transmembrane 6 superfamily, member 2 (TM6SF2) gene, represents another significant single nucleotide polymorphism (SNP) associated with NAFLD (Table 2). TM6SF2 is an endoplasmic reticulum (ER) transmembrane protein primarily expressed in hepatocytes, renal cells, and intestinal cells, playing a crucial role in the regulation of lipoprotein secretion [96]. The presence of the E167K variant disrupts the protein’s functionality, leading to a loss of its normal function and subsequently reducing the secretion of very low-density lipoprotein (VLDL) [97]. This variant has been linked to an increased susceptibility to NAFLD, hepatic steatosis, and advanced fibrosis (Table 2), while its association with inflammation remains inconclusive [98,99,100]. Although the impact of the E167K variant is relatively modest when compared to the PNPLA3 I148M variant, individuals harboring both the I148M and E167K variants exhibit a synergistic or additive effect, resulting in a twofold or cumulative risk of developing NAFLD [101]. These findings suggest the existence of gene–gene interactions contributing to the pathogenesis of the disease.

The E167K variant is present in lean individuals with NAFLD, in addition to those who are obese or overweight [102]. This observation underscores the specific involvement of the E167K variant in the development of NAFLD. Notably, the majority of studies examining “lean” NAFLD cases have predominantly focused on Asian populations [103], which aligns with the higher prevalence of the E167K variant in East Asian people. The minor T-allele frequency of this variant is more prevalent in East Asian populations (~34%) compared to European (~26%), Hispanic (~10%), and African (~6%) populations [104].

A study conducted by Liu et al. [105] demonstrated the potential impact of the TM6SF2 rs58542926 variant on fibrosis progression in NAFLD participants of European Caucasian descent. However, in contrast, Wong et al. [106] reported that the TM6SF2 rs58542926 variant did not contribute to the development of liver fibrosis or cirrhosis in Chinese individuals with NAFLD. These findings highlight the potential influence of genetic and ethnic variations on the association between the TM6SF2 variant and fibrosis progression in different populations.

In a recent exome-wide association study focusing on liver fat content, the TM6SF2 rs58542926 variant demonstrated a significant association with alanine aminotransferase (ALT) levels in both the Dallas Biobank and the Copenhagen Study. However, this variant did not show a statistically significant relationship with aspartate aminotransferase (AST) levels [44]. Nevertheless, these findings were not replicated in genome-wide association studies (GWASs) [107]. Conversely, certain GWASs have suggested a close association between the TM6SF2 rs58542926 variant and serum lipid levels [108,109].

In a meta-analysis conducted by Li et al., the TM6SF2 rs58542926 T-allele was confirmed as a risk factor for the susceptibility and development of NAFLD and its associated metabolic phenotypes in both adults and children [99]. Interestingly, the rs58542926 T-allele was found to be a protective factor for serum lipid levels. Notably, in this study, the risk of NAFLD associated with carrying the T-allele was higher in children compared to adults. The effect size of the rs58542926 T-allele was more pronounced in pediatric NAFLD than in adult NAFLD. Furthermore, their findings revealed that the rs58542926 variant was associated with the progression of steatosis, severe steatosis, fibrosis stages, and fibrosis progression in adults. However, there was no statistically significant difference observed in the fibrosis stages [99].

### 3.3. GCKR

The GCKR gene is responsible for encoding a glucokinase regulator that forms a complex with glucokinase and influences hepatic glucose storage and metabolism by directing its localization to the nucleus [110]. Any genetic variant that affects the functionality of the GCKR protein may contribute to the risk of NAFLD. In previous genome-wide association studies (GWAS) focusing on NAFLD, a common single nucleotide polymorphism (SNP) in the GCKR gene, namely rs780094, was identified and found to be associated with NAFLD [43]. This GWAS included 592 NAFLD patients with biopsy-proven cases from the NASH Clinical Research Network [43] and was the first to report the role of rs780094 in NAFLD. Furthermore, this study revealed a significant and robust association between rs780094 and lipid and glycemic traits. Subsequently, several genetic association studies have replicated and supported this association [48,111,112,113] (Table 2).

A meta-analysis conducted by Zain et al. examined the association of the GCKR rs780094 SNP with NAFLD, revealing a pooled effect of a 1.25-fold increased risk when comparing individuals carrying the T-allele with those carrying the C-allele [114]. This meta-analysis further demonstrated a significant association between rs780094 and NAFLD across different genetic models, including the dominant, recessive, and homozygote models [114].

The potential risk effect of the T-allele in NAFLD susceptibility has been observed in initial studies, which reported a significant association between the rs780094-T-allele and an increased risk of NAFLD. While two studies conducted by Yang et al. [113] and Gorden et al. [48] did not find a statistically significant association between the T-allele and the risk of NAFLD, the direction of the effect and the effect size demonstrated similar trends to the findings of the GOLD’s consortium [43]. It is worth noting that the pooled effective allele frequency exceeded 40% in each study population, emphasizing the substantial impact it can have on the risk of NAFLD.

In a comprehensive meta-analysis conducted by Li et al., which encompassed 25 studies with a total of 6598 cases and 19,954 controls, the objective was to precisely evaluate the association between GCKR polymorphisms and the risk of NAFLD [115]. The pooled estimates from the analysis revealed a significant predisposition to NAFLD in individuals carrying the T-allele of the GCKR rs780094 polymorphism [115].

### 3.4. LYPLAL1

The LYPLAL1 gene encodes a 26 kDa cytosolic protein known as lysophospholipase-like protein 1, which belongs to a subclass of the lysophospholipase family [116]. Several single nucleotide polymorphisms (SNPs) located near the LYPLAL1 gene have been found to exhibit a significant association with fat distribution, displaying a relatively sex-specific pattern [117]. In a large-scale genome-wide association study (GWAS) conducted by The Genetics of Obesity-Related Liver Disease Consortium, LYPLAL1 rs12137855 was identified as being associated with NAFLD in a cohort of 7177 adults of European ancestry [43]. This variant was found to be linked to steatosis as defined by computed tomography, as well as biopsy-proven NAFLD characterized by lobular inflammation and fibrosis [43].

In a study conducted by Sliz et al., the influence of genetic polymorphisms on the risk of NAFLD was investigated. It was observed that the metabolic effects associated with LYPLAL1 rs12137855-C were similar, albeit statistically less robust, to those observed with GCKR rs1260326-T. These findings suggest that LYPLAL1 may contribute to the regulation of circulating and hepatic triglyceride levels by influencing hepatic glucose metabolism, similar to the role played by GCKR. This hypothesis is supported by the findings of Ahn et al., who demonstrated that inhibiting LYPLAL1 leads to an increase in glucose production in the hepatocytes of humans, rats, and mice [118].

Nevertheless, conflicting results have been reported regarding the impact of LYPLAL1 rs12137855 on NAFLD steatosis. Several studies [111,118,119,120,121] do not provide evidence supporting the association between LYPLAL1 rs12137855 and NAFLD steatosis. As such, among all the genetic polymorphisms discussed, the influence of LYPLAL1 rs12137855 on the development of NAFLD remains the most contentious and subject to debate.

**Table 2:** Summary of commonly reported single nucleotide polymorphisms associated with susceptibility to MAFLD.

Gene	SNP	The Number of Patients	SNP Effects	Significance	Features	NAFLD Diagnosed by	Population	Reference(s)
PNPLA3	rs738409 C > G (I148M)	9515	Associated with NAFLD risk, steatosis and NASH	*p* > 0.001	Hepatic fat content exhibited a more than twofold increase in PNPLA3-148M homozygotes compared to individuals without this genetic variant.	H-MRS	African American; European American; Hispanic populations	[90]
		1117	Associated with steatosis and histological severity of NAFLD	*p* = 0.039 (steatosis); *p* < 0.001 (portal inflammation); *p* = 0.004 (NAS); *p* < 0.001 (fibrosis)	The presence of the G-allele in rs738409 was associated with the development of steatosis and greater histological severity of NAFLD. In pediatric patients, the high-risk G-allele in rs738409 was linked to an earlier onset of the disease.	Histologically	American population (894 adults/223 children)	[122]
		1092	Associated with steatosis and hepatocyte ballooning	*p* > 0.001 (steatosis); *p* = 0.006 (ballooning);	PNPLA3 rs738409 G-allele was correlated with liver steatosis and an elevated risk of progression from simple steatosis to NASH.	Histologically	American population	[48]
		126	Increased the risk for NAFLD	*p* < 0.001	The risk of NAFLD increased by 3.7-fold in subjects carrying the PNPLA3 GG genotype.	Ultrasonography	Hispanic children	[123]
		1709	Associated with NAFLD steatosis	*p* < 0.001	The G-allele was associated with elevated levels ALT, HOMA-IR *, and insulin.	MRI *	African American; Japanese American; Latino; Native Hawaiian; European American populations	[124]
		7176	Associated with NAFLD risk and steatosis	*p* < 0.001 (both)	N/A	CT, Histologically	European population	[43]
		417	Associated with steatosis	*p* < 0.0001	Individuals with the PNPLA3 GG genotype at rs738409 exhibited 2.7-fold higher liver fat content compared to those with the CC genotype.	Proton NMR *	Finnish population	[125]
		405	Associated with the ultrasonography-determined steatosis	*p* < 0.001	The 148M allele was linked to reduced levels of LDL-C * in patients with NAFLD.	Ultrasonography	Chinese population	[126]
		1027	Associated with NAFLD and moderate-to-severe steatosis	*p* = 0.006 (NAFLD); *p* = 0.001 (steatosis).	The G-allele of PNPLA3 rs738409 exhibited an association with NAFLD and a 1.09 IU/L increase in ALT levels.	Ultrasonography	Chinese children	[127]
		768	Associated with NAFLD	*p* = 0.00087	PNPLA3 GC and GG genotypes were significantly linked to an elevated risk of the disease.	Ultrasonography	Chinese population.	[119]
		4300	Associated with hepatic steatosis, and developed NAFLD and liver fibrosis	*p* < 0.001 (NAFLD)	Compared to CC homozygotes, GG homozygotes presented higher liver fat and liver fibrosis scores, despite having a better metabolic status (*p* < 0.05).	Ultrasonography	Chinese population	[128]
		879	Associated with NAFLD and insulin resistance	*p* = 0.004	The prevailing paradigm surrounding the PNPLA3 I148M (GG+GC) polymorphism indicates a positive correlation with elevated waist circumference, fasting insulin levels, HOMA-IR * scores, as well as higher concentrations of ALT and ferritin.	Ultrasonography	Normoglycaemic population	[129]
		270	Associated with NAFLD risk, steatosis, and fibrosis	*p* < 0.001 (NAFLD); *p* = 0.0003 (steatosis); *p* = 0.0445 (fibrosis)	Characterized by a pattern of steatosis, inflammation, and fibrosis, which are interconnected factors.	Histologically	German population (70 adolescents; 200 adult control cohort)	[130]
		515	Associated with liver steatosis, and fibrosis	*p* < 0.001 (steatosis); *p* < 0.001 (fibrosis)	The presence of the PNPLA3 risk allele exhibited heightened serum AST and ALT activities, with statistical significance observed at a p-values of less than 0.05.	Histologically (320 biopsied patients)	German population	[131]
		1326	Associated with steatosis, NAS * and fibrosis	*p* < 0.001 (NAFLD); *p* = 0.0016 (steatosis); *p* < 0.001 (NAS *)	The PNPLA3 risk allele was found to be linked with elevated levels of AST and ALT in individuals diagnosed with NAFLD.	Histologically and CT	Japanese population	[132]
		445	Associated with NAFLD risk, steatosis, fibrosis, and cirrhosis	*p* < 0.001 (NAFLD)	The ability to export VLDLs * from the liver is influenced by certain factors.	Ultrasonography	Italian population	[133]
		574	Associated with the severity of steatosis and fibrosis and the presence of NASH	95% CI = 1.04–1.76 (steatosis); CI = 1.12–2.04 (NASH)	The G-allele was observed to be disproportionately transmitted to children affected by the condition.	Histologically	Italian (253) and United Kingdom (321) population	[134]
		246	Associated with the risk of cirrhotic evolution	*p* < 0.001	In the NAFLD population, each copy of the G-allele was found to be associated with nearly a twofold increase in the risk of cirrhosis. Furthermore, individuals who were GG homozygous exhibited a tripled risk compared to those who were CC homozygous.	Histologically	Italian population	[95]
		1380	Associated with NAFLD risk, steatosis, NASH, fibrosis, cirrhosis and HCC *	*p* < 0.0001 (steatosis, NASH, fibrosis); *p* = 0.0007 (cirrhosis)	Such results are caused by the co-presence of the three at-risk variants: rs738409 C > G (PNPLA3 I148M), rs58542926 C > T (TM6SF2 E167K), and rs641738 C > T MBOAT7.	Histologically	European population	[135]
		470	Associated with NAFLD risk, steatosis and NASH	*p* < 0.001 (steatosis); *p* < 0.001 (lobular inflammation); *p* = 0.002 (ballooning)	The presence of specific features of steatohepatitis was found to be linked to the identified factor, but no significant associations were observed with liver fibrosis, anthropometry (body measurements), or insulin resistance.	Histologically	Belgian population	[136]
		285	Associated with NAFLD risk and NASH	*p* = 0.002 (NAFLD); *p* < 0.001 (NASH)	While the PNPLA3 genotype did not exhibit an association with the grade of steatosis, individuals with GG homozygosity had an increased likelihood of significant NASH activity and fibrosis.	Ultrasonography	Brazilian population	[137]
		342	Associated with NAFLD risk, NASH severity and fibrosis	*p* < 0.0001 (NAFLD); *p* < 0.0001 (NASH); *p* = 0.013 (fibrosis)	No associations were identified between the PNPLA3 genotype and simple steatosis or other histological parameters.	Histologically	Chinese, Indian and Malay	[138]
		365	Associated with the development of NAFLD and the severity of liver histology	*p* = 0.002 (NAFLD development); *p* < 0.005 (NAFLD severity)	Patients who possessed the PNPLA3 GG genotype exhibited higher levels of NAS * compared to those with the PNPLA3 CC genotype.	Histologically	Turkish population	[139]
		225	Associated with NAFLD and NASH risk, and fibrosis	*p* = 0.04 (NASH); *p* = 0.016 (fibrosis)	The GG genotype demonstrated an association with decreased platelet counts.	Histologically	Turkish population	[140]
		232	Associated with NAFLD, fibrosis but not steatosis	95% [CI] = 1.98–6.71 (NAFLD)	No significant associations were found between the GG genotype and body mass index, triglyceride levels, high- and low-density lipoprotein levels, or diabetes, as well as the steatosis grade (with a p-value greater than 0.05).	Histologically	Chinese population	[141]
		904	Associated with NAFLD in lean individuals	*p* = 0.003 (NAFLD)	Among individuals diagnosed with (NAFLD, a higher frequency of lean subjects (30.3%) carried the PNPLA3 rs738409 GG genotype compared to overweight (17.9%) and obese subjects (17.4%).	H-MRS *	Chinese population	[142]
		831	Associated with NAFLD and fibrosis, but not steatosis	*p* < 0.0001 (NAFLD); *p* = 0.011 (fibrosis)	The GG genotype was associated with elevated levels of AST (*p* = 0.00013), ALT (*p* < 0.0001), and ferritin (*p* = 0.014).	Histologically	Japanese population	[143]
		1461	Associated with NAFLD and NASH	*p* < 0.0001 (NAFLD, NASH)	Was also linked to hyaluronic acid levels, HbA1c * levels, and iron deposition in the liver.	Histologically	Japanese population	[144]
		339	Associated with NAFLD and fibrosis	*p* = 0.028 (NAFLD); *p* = 0.01 (fibrosis)	Within the NAFLD patient population, the frequency of CG+GG genotypes was significantly higher in individuals with advanced fibrosis	Ultrasonography	Korean population	[145]
		1363	Associated with NAFLD	*p* < 0.0001 (NAFLD)	Carriers of the rs738409-G-allele had a 1.19-fold increased risk for NAFLD and exhibited significantly lower levels of visceral and subcutaneous adiposity, body mass index, triglycerides, and insulin resistance compared to CC carriers.	Ultrasonography and CT	Korean population	[146]
		244	Associated with NAFLD, NASH risk.	*p* < 0.0005 (NAFLD); *p* < 0.05 (NASH)	N/A	H-MRS *	Indian population	[147]
		335	Associated with NAFLD risk	*p* = 0.04 (NAFLD)	The presence of the G-allele exhibited a significant association with higher levels of fasting insulin, HOMA-IR *, ALT, and AST values specifically among affected cases, while no such association was observed in the control group.	Ultrasonography	Asian Indian population	[148]
		200	Associated with NAFLD risk and steatosis	*p* < 0.05 (steatosis)	Patients carrying the G-allele demonstrated elevated levels of ALT, dyslipidemia, and insulin resistance.	Ultrasonography	Indian population	[149]
		306	Associated with NAFLD risk	*p* = 0.001 (NAFLD)	PNPLA3 gene polymorphism was found to be linked to higher levels of ALT.	Ultrasonography	Indian population	[150]
		207	Associated with NAFLD risk	*p* < 0.001 (NAFLD)	The PNPLA3 rs738409 gene polymorphism significantly increases the risk of NAFLD by up to four-fold in individuals with elevated triglyceride levels.	Ultrasonography	Indian population	[151]
		224	Associated with NAFLD, NASH, fibrosis, and cirrhosis.	*p* < 0.05	The GG genotype exhibited a 20.25-fold higher odds of developing NAFLD, as well as a 6.53-fold higher odds of experiencing non-alcoholic steatohepatitis (NASH).	Ultrasonography	Indian population	[152]
		144	Associated with MAFLD	*p* = 0.017	In a multivariable analysis, hypertriglyceridemia, BMI, and the PNPLA3 GG genotype were identified as factors associated with MAFLD.	CT, MRT	Chinese population.	[153]
		143	Associated with NAFLD	*p* = 0.002	The presence of PNPLA3 risk alleles impairs the response to dietary interventions in individuals diagnosed with NAFLD.	Ultrasonography	German population	[154]
		525	Associated with NASH and fibrosis	*p* = 0.008 (NASH); *p* = 0.020 (fibrosis)	The PNPLA3 genotype showed an association with the HOMA-IR * and insulin resistance in adipose tissue.	Histologically	Korean population	[155]
		211	Associated with NAS * (NAFLD Activity Score)	NAS: ≤2 vs. ≥3, *p* = 0.667; ≤4 vs. ≥5, *p* = 0.034)	The PNPLA3 genotype was found to have a partial impact on the NAFLD activity score.	Histologically	Japanese population	[156]
		4804	Associated with steatosis	*p* = 0.01	The presence of PNPLA3 variants was found to be associated with elevated levels of ALT.	Ultrasonography	Non-Hispanic white, non-Hispanic black, and Mexican American participants in the US population	[157]
		797	Associated with NAFLD	*p* = 0.008	PNPLA3 variants may contribute to the susceptibility of NAFLD in obese individuals across various ethnic groups.	Ultrasonography	Chinese children	[111]
		307	Associated with NAFLD	*p* < 0.01	No significant effect modification was observed with BMI.	FibroScan	Mexican population	[120]
		382	Associated with NAFLD, and fibrosis	*p* = 0.0044 (NAFLD); *p* = 0.0272 (fibrosis)	Individuals with the PNPLA3 GG genotype had a significantly increased risk (3.29-fold) of developing NAFLD compared to those with the CC genotype.	Histologically	Brazilian population	[158]
		349	Increased the risk of NAFLD	*p* = 0.29	Although the presence of the GG genotype showed a 1.39 times increased risk of NAFLD, this association did not reach statistical significance.	Histologically and Ultrasonography	Turkey population	[159]
GCKR	rs780094 C > T	1092	Was not associated with NAFLD	*p* > 0.05	The GCKR SNP rs780094 exhibited a significant association with elevated serum triglyceride levels (*p* = 0.04).	Histologically	American	[48]
		270	Associated with NAFLD risk, steatosis, and especially fibrosis	*p* = 0.0281 (NAFLD); *p* = 0.0275 (fibrosis)	In individuals with the rs738409 G/G genotype, proteome profiling analysis revealed a reduction in the levels of GCKR protein and a downregulation of the retinol pathway.	Histologically	German population (70 adolescents; 200 adult control cohort)	[130]
		7176	Associated with NAFLD risk and steatosis	*p* < 0.001 (NAFLD risk); *p* = 0.01 (steatosis)	N/A	CT; Histologically	European population	[43]
		4804	Associated with steatosis	*p* = 0.03	It was associated with a high level of ALT.	Ultrasonography	Non-Hispanic white, non-Hispanic black, and Mexican American participants in the US population	[157]
		366	Associated with the severity of liver fibrosis	*p* < 0.001	Associated with higher serum triglyceride levels (*p* = 0.02).	Histologically	Italian population	[160]
		797	Associated with NAFLD	*p* = 0.008	Associated with higher mean serum ALT concentration.	Ultrasonography	Chinese children	[111]
		620	Associated with NAFLD	95% CI: 1.14–1.28 (NAFLD)	Demonstrated an association with specific dietary habits, such as the consumption of soda, eggs, and soybean.	Ultrasonography	Uyghur population	[121]
		342	Associated with NAFLD, NASH, and fibrosis	*p* = 0.013 (NAFLD); *p* = 0.012 (NASH); *p* = 0.038 (fibrosis)	The combined effect of GCKR and adiponutrin rs738409 indicated a substantially increased risk of NAFLD (*p* = 0.010).	Histologically	Malaysian (Malay, Chinese, and Indian) population	[112]
		903	Associated with NAFLD	*p* = 0.0072	The T-allele of GCKR rs780094 showed a significant association with an elevation in fasting triglyceride levels.	Ultrasonography	Chinese population	[113]
TM6SF2	rs58542926 C > T	768	Associated with NAFLD	*p* = 0.0016	The T-allele of TM6SF2 rs58542926 showed a higher prevalence among subjects diagnosed with NAFLD.	Ultrasonography	Chinese population	[119]
		515	Associated with NAFLD risk and steatosis but not fibrosis	*p* = 0.003 (steatosis)	Associated with significantly increased AST but not ALT.	Histologically (320 biopsied patients)	German population	[131]
		445	Associated with NAFLD risk	*p* = 0.008 (NAFLD)	Affects the liver’s ability to export very low-density lipoproteins (VLDLs).	Ultrasonography	Italian population	[133]
		1380	Associated with NAFLD risk, steatosis, NASH, fibrosis, cirrhosis and HCC *	*p* < 0.0001 (steatosis, NASH, fibrosis); *p* = 0.0007 (cirrhosis)	Such results are caused by the co-presence of the 3 at-risk variants: rs738409 C > G (PNPLA3 I148M), rs58542926 C > T (TM6SF2 E167K), and rs641738 C > T MBOAT7.	Histologically	European population	[135]
		3260	Associated with NAFLD	*p* = 0.02	No significant effect on inflammation was observed for the rs58542926 T-allele.	Histologically	International	[161]
		361	Associated with NAFLD, steatosis and disease severity	*p* = 0.038 (NAFLD)	rs58542926 was not associated with levels of liver enzymes, lobular inflammation and fibrosis.	Ultrasonography and Histologically	Argentina population	[107]
		300	Associated with liver fat	*p* < 0.05	Individuals with this variant exhibit preserved insulin sensitivity in relation to processes such as lipolysis and hepatic glucose production, and they do not typically experience hypertriglyceridemia	H-MRS	Finnish population	[162]
		143	Associated with NAFLD	*p* = 0.041	The presence of TM6SF2 risk alleles hinders the response to dietary interventions in individuals diagnosed with NAFLD.	Ultrasonography	German population	[154]
		1010	Associated with steatosis	*p* < 0.0001 (steatosis)	It is associated with higher levels of ALT and lower levels of total cholesterol, low-density lipoprotein cholesterol, triglycerides, and non-high-density lipoprotein cholesterol.	Ultrasonography	Italian children	[163]
		878	Associated with steatosis	*p* = 0.002	Carriers of the TM6SF2 167K variant have a threefold increased risk of developing hepatic steatosis, which often manifests early in life.	Ultrasonography	Italian children	[164]
		957	Associated with NAFLD risk, steatosis and fibrosis	*p* = 0.05 (NAFLD); *p* < 0.05 (steatosis)	Associated with high HFF% in Caucasian and African American populations, with high ALT levels in Hispanic populations and with a more favorable lipoprotein profile in Caucasian and Hispanic populations.	MRI * and Histologically	Caucasian, African, American, and Hispanic children and adolescents	[47]
		1074	Associated with NAFLD risk, steatosis, NASH, advanced hepatic fibrosis	*p* = 0.0008 (NAFLD); *p* < 0.001 (steatosis); *p* = 0.039 (NASH); *p* = 0.0074 (fibrosis)	Carriage of the TM6SF2 variant does not appear to further increase HCC * risk independently of its effect on fibrosis stage.	Histologically	Caucasian and European populations	[105]
		316	Associated with NAFLD risk and steatosis	*p* = 0.003 (NAFLD); *p* = 0.023 (steatosis)	Associated with increased ALT but no other clinical parameters, such as AST, ALP * and lipids.	FibroScan	Chinese population	[101]
		768	Associated with NAFLD risk	*p* = 0.0007	TM6SF2 167K allele was associated with NAFLD after adjustment for age, sex, body mass index and status of diabetes.	Ultrasonography	Chinese population	[165]
		1201	Associated with NASH and fibrosis	*p* < 0.05	Associated with more severe steatosis, necroinflammation, ballooning, and fibrosis.	Histologically	Italian, Finnish, and Swedish populations	[166]
		525	Associated with NASH and fibrosis	*p* = 0.008 (NASH); *p* = 0.020 (fibrosis)	Even after adjustment for metabolic risk factors, rs58542926 increased the risk of NASH and significant fibrosis.	Histologically	Korean population	[155]
		503	Associated with NAFLD risk	*p* = 0.0004	The presence of rs58542926 variant in the TM6SF2 gene exhibited a significant association with NAFLD, indicating a 2.7-fold higher risk of developing the condition.	Ultrasonography	South Indian and North-East Indian populations	[167]
		285	Was not associated with NAFLD risk	*p* = 0.78	The presence of the T-allele was not found to be associated with NAFLD or NASH, and it did not show any association with histological features related to these conditions.	Ultrasonography	Brazilian population	[137]
		144	Was not associated with NAFLD risk	*p* > 0.05	There was no association between rs58542926 and liver steatosis (*p* = 0.62), ballooning (*p* = 0.14), lobular inflammation (*p* = 0.99) and fibrosis (*p* = 0.89)	CT, MRT	Chinese population	[153]
		211	Was not associated with NAS	*p* > 0.05	The TM6SF2 genotype did not affect the NAFLD activity score (≤2 vs. ≥3, *p* = 0.867; ≤4 vs. ≥5, *p* = 0.936).	Histologically	Japanese population	[156]
LYPLAL1	rs12137855 C > T	7176	Associated with NAFLD risk and steatosis	*p* < 0.001; (NAFLD risk)	C-allele was associated with CT-defined steatosis and biopsy-proven NAFLD.	CT; Histologically	European	[43]
		797	Was not associated with NAFLD	*p* > 0.05	N/A	Ultrasonography	Chinese children	[111]
		307	Was not associated with NAFLD	*p* > 0.05	N/A	FibroScan	Mexican population	[120]
		620	Was not associated with NAFLD	*p* > 0.05	N/A	Ultrasonography	Uyghur population	[121]

* LDL-C—low-density lipoprotein cholesterol; HOMA-IR—homeostasis model assessment of insulin resistance; NAS—NAFLD activity score; VLDLs—very-low-density lipoproteins; HCC—hepatocellular carcinoma; HbA1c—hemoglobin A1C; ALP—alkaline phosphatase; CT—computerized tomography. MRI - magnetic resonance imaging; H-MRS—proton magnetic resonance spectroscopy; Proton NMR—proton nuclear magnetic resonance.

## 4. The Influence of MAFLD-Associated Polymorphisms on the Severity of COVID-19

The COVID-19 pandemic has brought to light the association between NAFLD and increased susceptibility to severe SARS-CoV-2 infection [36,37,38,168].

Consequently, it has been hypothesized that genetic variants associated with NAFLD may indirectly influence the severity of COVID-19 infection. This intriguing hypothesis has motivated investigations into candidate genes through association studies. One such study utilized the UK Biobank dataset to develop a genetic risk score for NAFLD, considering the combined effects of variants involved in hepatic fat accumulation (PNPLA3-TM6SF2-MBOAT7-GCKR) [51]. Building upon this knowledge, Valenti et al. examined the impact of this NAFLD-genetic risk score on the susceptibility to COVID-19 and observed a trend suggesting that the rs738409 variant conferred protection against COVID-19 [51].

Grimaudo et al. conducted a study that revealed a significant association between the rs738409 G-allele and severe COVID-19 outcomes in patients aged 65 years or younger [50].

In contrast, Innes et al. reported a striking inverse association between rs738409 and the severity of COVID-19 outcomes in a cohort of 1585 participants from the UK Biobank [52]. Their findings indicated that the rs738409-G-allele was independently associated with a reduced risk of COVID-19 hospitalization and mortality. Importantly, this protective effect persisted even after adjusting for major demographic factors and underlying metabolic and liver co-morbidities [52].

From a functional perspective, the observed association between lipid metabolism and the immune response to COVID-19 could be attributed to various factors. For instance, retinoids are stored as retinyl esters in hepatic mesenchymal cells and adipose tissue, where the PNPLA3 gene is expressed. When the need arises, retinoids are mobilized to extrahepatic tissues, where they play a crucial role in stimulating the production of interferon type 1, a potent cytokine response to viral infections [169]. Conversely, certain risk factors associated with severe COVID-19, such as obesity and liver disease [170], are known to be linked to decreased retinoid levels and impaired retinoid signaling. This impairment could potentially limit the availability of retinoids during infection. Moreover, individuals with the rs738409 G-allele may exhibit a lower ratio of omega-6 to omega-3 polyunsaturated fatty acids, which has been implicated in modulating inflammation and providing protection against cytokine storm syndrome [171].

Furthermore, Innes et al. [52] conducted a comprehensive meta-analysis encompassing three distinct data sources to explore the potential relationship between the rs738409 variant and COVID-19. The study incorporated data from the FinnGen study, which consisted of 83 individuals with COVID-19 hospital admissions and 274 SARS-CoV-2-positive patients without hospital admission. Additionally, the Geisinger Health System dataset included 854 subjects of European Ancestry, with 165 individuals experiencing COVID-19 hospitalization and 689 SARS-CoV-2-positive patients without hospital admission. Lastly, the study by Grimaudo et al. contributed data from a total of 383 COVID-19 patients [50]. The pooled analysis of these aforementioned data sources revealed that the presumed protective effect of the G “NASH-risk allele” on COVID-19 morbidity and mortality could not be definitively confirmed. Nevertheless, there was a discernible trend suggesting an association with a reduced risk of COVID-19 hospitalization and severe disease, although this trend did not reach statistical significance [52].

Similarly, Bianco et al. conducted a study examining the potential impact of the rs738409 G-allele on COVID-19 outcomes. Their findings indicated that this allele exhibited a tendency not only to be associated with protection against COVID-19 but also with lower levels of C-reactive protein, despite higher ALT and lower albumin levels in severe COVID-19 patients of European ancestry [53].

Currently, the body of research investigating the influence of genetic polymorphisms associated with NAFLD on the progression and severity of coronavirus disease remains limited (Table 3). However, these studies shed light on the potential interplay between NAFLD-related genetic variants and the course of COVID-19. Further investigations are warranted to elucidate the underlying mechanisms and determine the clinical implications of these associations.

## 5. How Do MAFLD-Associated Polymorphisms Affect Gene Expression in Different Tissues?

Understanding the causality between genotypes and phenotypes provides valuable insights into the genes and their interactions that contribute to the expression of specific traits in organisms. This is particularly relevant for comprehending complex traits that result from the combined effects of multiple genes and environmental factors.

Currently, the investigation of expression quantitative trait loci (eQTLs) represents a prominent and extensively explored avenue for understanding the functional consequences of genetic variation [172]. Numerous genetic studies focusing on gene expression have successfully identified thousands of eQTLs across diverse tissue types, encompassing a large proportion of human genes.

The comprehensive collection of eQTLs serves as a valuable tool for exploring the underlying molecular mechanisms of prevalent genetic disorders [173,174].

Our current knowledge of gene expression genetics heavily relies on the identification of eQTLs, which represent the associations between gene expression levels and specific genotypes at particular genomic loci. Genome-wide investigations of eQTLs have revealed that these loci contribute significantly to the variation in gene expression, with certain genes exhibiting up to 90% of their expression variation attributable to nucleotide variants.

**Figure 2:**
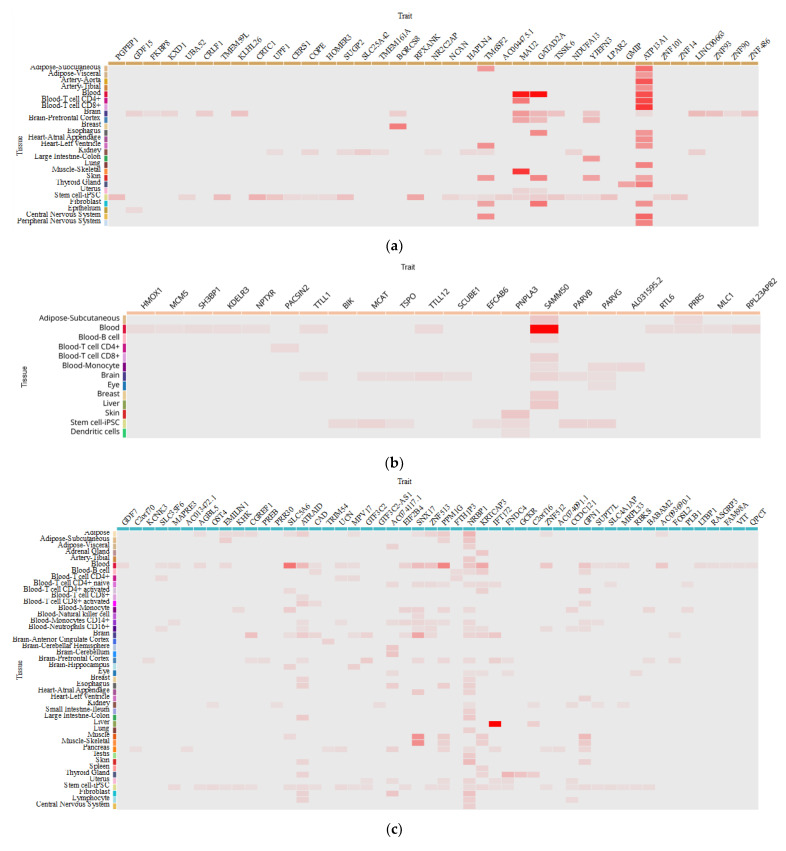

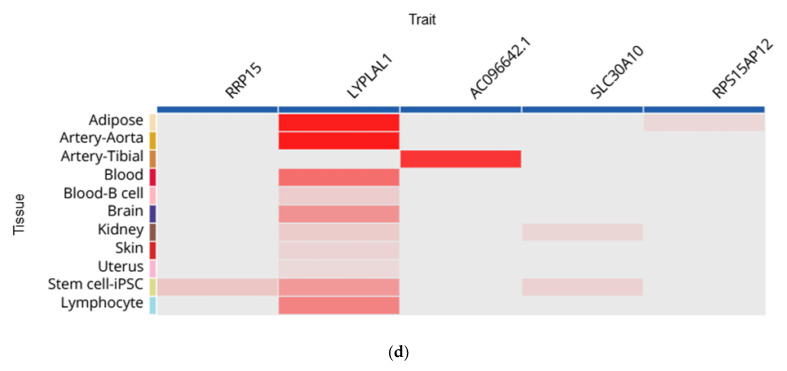
The heatmap plot illustrates the distribution of molecular traits associated with various tissue/cell types and compares them to eQTL-associated traits (genes). Each row corresponds to a unique tissue or cell type, and each column represents a distinct eQTL-associated trait (gene). The color of each grid cell indicates the median P-value of the eQTLs associated with the specific tissue and trait combination. Overview of eQTL for TM6SF2 rs58542926 (**a**) 47 tissues and 25 traits; PNPLA3rs738409 (**b**) 13 tissues and 22 traits; GCKR rs780094 (**c**) 44 tissues and 50 traits; GCKR rs780094 (**d**) 11 tissues and 5 traits.

By utilizing the eQTL database available at http://www.mulinlab.org/qtlbase/index.html (accessed on 15 June 2023), we can examine whether the four aforementioned SNPs exhibit eQTL effects in various tissue types. Selected findings are presented in Table 4 for reference. Notably, these data shed light on the potential impact of single-nucleotide polymorphisms within the GCKR (rs780094), PNPLA3 (rs738409), TM6SF2 (rs58542926), and LYPLAL1 (rs12137855) genes on gene expression patterns in immune cells and blood (Figure 2). This implies that these genetic variants could potentially influence the immune response against infectious diseases, including COVID-19.

In light of these findings, it is evident that these genetic polymorphisms hold promise as prospective targets for future research endeavors. Their potential influence on gene expression, particularly in immune cells and blood, suggests their potential involvement in modulating the immune response to infectious diseases, including COVID-19. As such, investigating the functional implications of these genetic variants could provide valuable insights into disease susceptibility, pathogenesis, and therapeutic strategies [175,176,177,178,179]. Therefore, further exploration of these genetic polymorphisms is warranted to deepen our understanding of their role and potentially identify novel avenues for therapeutic interventions.

## 6. Discussion

It would be interesting to investigate the potential synergistic effects of these genetic polymorphisms in shaping the complex interplay between NAFLD susceptibility and COVID-19 outcomes. One could think of conducting comprehensive studies that take into account the combined influence of multiple risk-associated variants considering both additive and interactive effects. This could provide a more accurate representation of the genetic landscape contributing to COVID-19 severity in individuals with NAFLD.

Furthermore, it is worth considering the implications of these genetic variants on the intricate pathways governing lipid metabolism, immune response modulation, and cytokine release, all of which have been implicated in both NAFLD and COVID-19 pathogenesis. Investigating how these pathways intersect could shed light on potential therapeutic targets or strategies to mitigate the impact of these genetic variants on disease severity. This may involve delving into advanced molecular techniques such as transcriptomics, proteomics, and metabolomics to decipher the molecular underpinnings of these variants’ effects. Additionally, creating comprehensive gene profiling tools that consider the collective influence of these genetic variants, along with clinical and demographic factors, could provide a powerful predictive tool for identifying individuals at risk of severe COVID-19 outcomes.

The available genetic data thus far do not provide strong evidence for a significant predisposition conferred by MAFLD to the development of severe COVID-19. However, the COVID-19 pandemic has underscored the importance of obtaining a comprehensive understanding of not only the associations attributed to the PNPLA3 gene with liver-related traits but also the intricate protein interactions, active protein ligands, and, crucially, the accurate and comprehensive assessment of the variant pleiotropic effects.

Considering these factors, we advocate for a comprehensive approach to evaluating the polymorphisms of the host’s genetic determinants, particularly those associated with susceptibility to MAFLD. This approach should aim to develop gene profiling tools that can support early prediction at the individual level during the course of COVID-19. If confirmed as determinants of disease severity, these host polymorphisms could enable the identification of vulnerable populations or patients at higher risk for severe outcomes, thereby facilitating improved the diagnosis, treatment, and prognosis of COVID-19 [180,181].

## 7. Conclusions

In conclusion, this review underlines the intricate relationship between NAFLD-associated genetic variants and COVID-19 severity. As we delve deeper into understanding the molecular mechanisms and interactions between these variants, we pave the way for potential targeted interventions and predictive strategies to effectively manage COVID-19 outcomes in individuals with MAFLD.

## Figures and Tables

**Table 3:** The impact of MAFLD-associated polymorphisms on the severity course of COVID-19.

Gene	SNP	The Number of Patients	SNP ’s Effect	Significance	Features	Patient Cohorts	References
PNPLA3	rs738409 (C > G) (I148M)	383	Associated with an increased risk of severe COVID-19 outcomes	*p* = 0.035 (GG genotype)	Individuals harboring a GG genotype in the PNPLA3 gene may exhibit inherent upregulation of the NLRP3 inflammasome, rendering them more susceptible to tissue damage upon infection with SARS-CoV-2.	Italian populations	[50]
		1460	Was not associated with the risk of severe COVID-19	*p* > 0.1	There appears to be an inclination towards protection against COVID-19 when considering the aforementioned genotype. These findings imply that the genetic inclination towards hepatic fat accumulation does not independently heighten the susceptibility to severe COVID-19. Moreover, this indicates that MAFLD does not assume a causal role in this particular condition.	UK populations	[51]
		1585	Associated with a lower risk of COVID-19 hospitalization and death	*p* = 0.027 (hospitalization); *p* = 0.037 (death)	On average, the presence of each additional G-allele was associated with a notable decrease of 21% in the likelihood of COVID-19 hospitalization and a further decrease of 25% in the likelihood of COVID-19-related mortality.	UK populations	[52]
		1397	Was not associated with the risk of severe COVID-19 in hospitalized patients	*p* = 0.46	Intriguingly, a genetic predisposition to accumulate fat in the liver may paradoxically confer protection during the course of COVID-19.	Hospital-based Fondazione IRCCS Ca’ Granda cohort	[53]

**Table 4:** EQTL data for SNPs association in tissues.

Trait	CHR	Effective Allele	Tissue	Effect Size	*p*-Value	Population	Sample Size
GCKR rs780094							
AC074117.1	2	C	Fibroblast	−0.206732	3.23 × 10^−9^	MIX	483
ATRAID	2	T	Blood	−0.20743	2.2 × 10^−13^	MIX	2765
ATRAID	2	C	Blood-T cell CD8+ activated	0.130598	0.00000114	EAS	416
ATRAID	2	C	Lymphocyte	0.163788	0.0000466	EUR	368
EIF2B4	2	C	Blood	−0.00922729	0.0000631	MIX	5257
EMILIN1	2	T	Adipose-Subcutaneous	0.24	0.00000333	EUR	770
GPN1	2	C	Blood-T cell CD4+ activated	0.188994	0.00000277	EAS	416
KRTCAP3	2	T	Blood	−0.237548	6.3 × 10^−17^	MIX	2765
KRTCAP3	2	C	Blood-T cell CD4+ activated	0.234009	0.0000181	EAS	416
NRBP1	2	C	Lymphocyte	−0.141265	0.00000365	EUR	368
NRBP1	2	C	Blood	−0.013931	2.04 × 10^−12^	MIX	5257
NRBP1	2	T	Blood	0.153229	0.000000044	MIX	2765
NRBP1	2	NA	Blood-Monocyte	5.524902	6.21 × 10^−8^	EUR	432
PPM1G	2	C	Adipose	−0.331022	0.0000007	EUR	434
SLC5A6	2	T	Blood	0.165322	4.7 × 10^−9^	MIX	2765
SLC5A6	2	NA	Blood-Monocyte	0.233295	0.00000113	MIX	696
SNX17	2	C	Blood-Monocytes CD14+	−0.03898	0.00000409	MIX	197
SNX17	2	T	Blood	0.141788	0.00000034	MIX	2765
SNX17	2	NA	Blood-Monocyte	4.2447721	0.0000286	EUR	432
ZNF512	2	C	Blood	0.0147365	7.47 × 10^−8^	MIX	5257
AC074117.1	2	C	Fibroblast	−0.206732	3.23 × 10^−9^	MIX	483
ATRAID	2	T	Blood	−0.20743	2.2 × 10^−13^	MIX	2765
ATRAID	2	C	Blood-T cell CD8+ activated	0.130598	0.00000114	EAS	416
ATRAID	2	C	Lymphocyte	0.163788	0.0000466	EUR	368
PNPLA3 rs738409							
SAMM50	22	G	Blood-T cell CD8+	−3.90455	9.44 × 10^−5^	EUR	283
PNPLA3	22	G	Skin	−0.13586	1.54 × 10^−6^	MIX	605
SAMM50	22	C	Blood	−0.07719	5.67 × 10^−106^	MIX	5257
SAMM50	22	G	Adipose-Subcutaneous	0.188769	9.6 × 10^−7^	EUR	385
AL031595.2	22	G	Blood-Monocyte	0.220815	0.00188	EAS	416
TM6SF2 rs58542926							
ATP13A1	19	G	Blood	0.442548	3.13 × 10^−8^	EUR	121
ATP13A1	19	T	Blood-T cell CD4+	5.639089	1.71 × 10^−8^	EUR	293
ATP13A1	19	T	Blood-T cell CD8+	5.943743	2.79 × 10^−9^	EUR	283
BORCS8	19	T	Breast	−0.27489	4.48 × 10^−6^	MIX	396
GATAD2A	19	C	Blood	−0.03402	5.07 × 10^−15^	MIX	5257
GATAD2A	19	T	Blood	0.163165	3.74 × 10^−6^	MIX	670
MAU2	19	T	Blood-T cell CD4+	−4.67124	2.99 × 10^−6^	EUR	293
MAU2	19	T	Blood	−0.19358	6.75 × 10^−14^	MIX	670
MAU2	19	T	Blood	−0.18721	2.07 × 10^−7^	EUR	369
TM6SF2	19	T	Adipose-Subcutaneous	0.32323	7.09 × 10^−5^	MIX	581
YJEFN3	19	T	Large Intestine-Colon	0.200617	7.71 × 10^−5^	MIX	318
LYPLAL1 rs12137855							
LYPLAL1	1	C	Lymphocyte	−0.122143	0.00085	EUR	368
LYPLAL1	1	NA	Blood	−0.220481	0.000305	EUR	240
SLC30A10	1	NA	Stem cell-iPSC	−0.143156	0.0313	EUR	215
LYPLAL1	1	T	Blood-B cell	−2.04427	0.0409	EUR	45
RPS15AP12	1	T	Adipose	0.146055	0.0723	EUR	434
AC096642.1	1	T	Artery-Tibial	−0.14702	1.58 × 10^−5^	MIX	584

## Data Availability

Data are contained within the article.
